# Joint Action against Small-Pox

**Published:** 1896-05-23

**Authors:** 


### The Hospital

An Institutional Journal of

The Medical Sciences and Hospital Administration, with

Vol. XX.—No. 504] AN EXTRA NURSING SUPPLEMENT. [May 23, 1896.

### Joint Action against Small-pox.

The troubles of Gloucester are having a considerable influence in other ways besides merely stimulating that craving for safety which shows itself by a general rush to vaccination. In many parts of the country sanitary authorities are being led to furbish up their armour and reconsider their means of defence against a possible outbreak of small-pox, and, among other things which are being brought home to them, is the desirability of arranging for combinations among contiguous districts in the matter of small-pox hospitals. If it were only for the sake of facilitating the discovery of sites for such buildings combination would be worth while, for the site difficulty is a real trouble.

This question of hospital accommodation is of especial importance in the cases where moderate-sized urban districts are surrounded by small rural ones. In such cases the advantages of both the urban and the rural districts combining for hospital purposes are obvious, for it almost always happens that a town is the centre to which the roads of the district converge, and that the site which is most convenient for the town is also the most generally convenient for the surrounding area.

The desirability of combining while there is yet time, before the towns have provided themselves with hospitals and so cut up the country districts into straggling and disconnected areas, is well illustrated by what is going on at Halifax, where a meeting of representatives of the various local authorities within the Union was held last week to consider the desirability of conjoint action in the building of a small-pox hospital. According to the report in the *Halifax Courier* it would appear that Halifax, a town with nigh upon 100,000 inhabitants, possesses a certain amount of hospital accommodation for small-pox, but of such a kind that, in the words of the Town Clerk, "The Local Government Board have condemned it as a small-pox hospital, lock, stock, and barrel." The out-districts also, although they have made some efforts in recent times to provide accommodation for such cases as have occurred, clearly have done so in a most perfunctory and imperfect manner. Like certain parties in the town, who maintain that the buildings which have been provided are sufficient, so in these little out-districts there are always to be found groups of councillors who imagine that the hire of a cottage here, or the erection of a temporary hospital there, is sufficient for all purposes. But here is what Dr. Ainley, the medical officer of health, says: "The whole point was to be efficiently provided. Some time ago the Greetland Council took two cottages on a public roadside for small-pox hospital purposes, and anyone going past might have taken the disease. What had Sowerby Bridge done? Taken a cottage with public highways on both sides of it, and there the patients and nurses had to live and sleep together. He was amazed Sowerby Bridge men now talked as they did. Their position was something disgraceful. 'We are prepared,' they said. Good heavens! In no sense whatever were they prepared. They ought to be thankful and delighted that this combination scheme was being propounded. He had seen the outside districts in the Union, and not one of them was prepared, as it should be, to deal with small-pox." Alderman Sugden also stated that not one authority in the Union could in regard to provision for small-pox cases satisfy the Local Government Board.

Halifax, however, is by no means a solitary example of the difficulties which surround sanitary authorities in their isolated attempts to provide for small-pox. Fortunately this is a disease which usually bears transportation fairly well, and in regard to which therefore there is everything to be gained by the combination of districts for hospital purposes. Moreover, there is this very important fact to be remembered, that the districts are not entirely free agents in the matter. Under the Isolation Hospitals Act if they do not make proper provision the County Council can at any time intervene and compel the formation of combined districts, without, however, the advantage of combining with such towns as are county boroughs. Under these circumstances the suggestion that the rural should, where it is possible, combine with the urban districts is worthy of the most serious consideration. The natural centre of a district is the town which lies in its midst, and the site for a hospital which is convenient for the town is almost sure to be the one which is most convenient for the major part of the country which surrounds it. In this then, as in other affairs, the principle of "give and take" should be adopted. Towns lack sites on which to build isolation hospitals, sites which country places can more easily supply; while rural authorities greatly lack money with which to build them, a want not so acutely felt in towns, while both will find that they can easily do in combination what is most difficult to perform alone. In every way then joint small-pox hospitals are to be commended. There should, however, be no delay. Once let the towns obtain well-fitted independent hospitals and they certainly will not join in any combination, but will leave the scattered rural districts to do the best they can.

